# *Moringa oleifera* leaf extract enhances rumen degradability and modifies methanogen communities *in vitro*

**DOI:** 10.3934/microbiol.2025043

**Published:** 2025-12-16

**Authors:** Amr E. El-Nile, Marwa F. A. Attia, Mahmoud A. Elazab, Mohamed N. El-Gendy, Ahmed E. Kholif, Raed A. Aburawash, Elsayed E. Hafez, Sobhy M.A. Sallam

**Affiliations:** 1 Department of Livestock Research, Arid Land Cultivation Research Institute, City of Scientific Research and Technological Applications, New Borg El Arab, 21934, Alexandria, Egypt; 2 Animal Production Research Institute, Agricultural Research Center, Giza 12618, Egypt; 3 Department of Animal Sciences, North Carolina Agricultural and Technical State University, Greensboro, NC 27411, USA; 4 Dairy Science Department, National Research Centre, 33 Bohouth St. Dokki, Giza, Egypt; 5 Plant Protection and Biomolecular Diagnosis Department, Arid Lands Cultivation Research Institute, City of Scientific Research and Technological Applications, New Borg El-Arab City, 21934, Alexandria, Egypt; 6 Department of Animal and Fish Production, Faculty of Agriculture, Alexandria University, 21545, Alexandria, Egypt; 7 Department of Animal and Poultry Production, College of Agriculture and Food, Qassim University, Buraydah 51452, Al-Qassim, Saudi Arabia

**Keywords:** *Moringa oleifera* leaf extract, *in vitro* gas production, rumen degradability, methane emissions, methanogen community

## Abstract

The development of innovative feed resources for livestock is crucial for ensuring nutrient adequacy while reducing greenhouse gas emissions. We aimed to evaluate the effects of *Moringa oleifera* leaf extract (ML) supplementation on in vitro nutrient degradability, net gas production (GP), ruminal fermentation, methane (CH_4_) emissions, and methanogen community structure using a semi-automated in vitro gas production system. Methanogen-specific 16S rRNA genes were amplified through nested PCR and then sequenced with Sanger sequencing. Microbial analyses were conducted using 16S rRNA sequencing. A basal diet (50% concentrate and 50% forage) was incubated in vitro for 24 h as a control (no additives) and compared to diets supplemented with ML at 1.0, 2.0, and 3.0 mL/100 g dry matter (DM), designated ML1, ML2, and ML3, respectively. GC-MS profiling of ML revealed that glycerin (82.08%), unsaturated fatty acid derivatives such as linoleic acid, and minor bioactive sulfur- and nitrogen-containing compounds (e.g., L-cystathionine, homocysteine derivatives) were the major constituents. These compounds exert antimicrobial, membrane-disrupting, and redox-modulating effects, which provide the basis for the proposed mechanisms by which ML influences rumen fermentation and methanogenesis. Supplementation with ML significantly reduced net GP (linear, P < 0.001; quadratic, P = 0.002) and CH_4_ production (linear, P = 0.033) across all levels. Similarly, truly degradable dry matter (TDDM; linear, P = 0.038) and truly degradable organic matter (TDOM; linear, P = 0.016) decreased, whereas the partitioning factor increased with ML1 and ML2 supplementation (quadratic, P = 0.002). Ruminal pH and ammonia nitrogen (NH_3_-N) concentrations remained unaffected. However, ML treatments reduced total volatile fatty acids (linear, P = 0.009; quadratic, P = 0.003) and butyrate concentrations (linear, P < 0.001). Acetate and propionate concentrations were reduced by ML1 and ML2 (quadratic, P = 0.005). In contrast, ML3 increased isobutyrate (linear, P = 0.004; quadratic, P = 0.012) and isovalerate (linear, P = 0.023; quadratic, P = 0.012) levels. Protozoal enumeration showed that Diplodinium spp. counts decreased with ML (linear, P = 0.008), while Epidinium spp. counts were reduced by ML1 (quadratic, P = 0.048). Phylogenetic analysis of 16S rRNA gene sequences indicated that ML supplementation altered the rumen methanogen community, with distinct shifts toward *Methanobrevibacter smithii* and *M. woesei* in ML2 and ML3, respectively. These findings suggest that ML selectively inhibits methanogenic archaea, potentially contributing to reduced CH_4_ emissions and altered fermentation profiles.

## 1. Introduction

The livestock industry, particularly ruminant livestock, is a significant contributor to greenhouse gas emissions, which exacerbates global warming. Methane (CH_4_), a potent greenhouse gas with a global warming potential approximately 28 times greater than carbon dioxide (CO_2_) over a 100-year period, is a major contributor. Annually, ruminants produce 81–92 million metric tons of CH_4_, accounting for 23–27% of global anthropogenic CH_4_ emissions, equivalent to emissions from human activities. Within the agricultural sector, cattle contribute 49.10% of enteric CH_4_ emissions, followed by buffalo, goats, sheep, and other ruminants at 42.80%, 5.38%, 2.59%, and 0.73%, respectively. To mitigate CH_4_ emissions from ruminants, researchers have explored various strategies, including the use of amino acids, organic acids, essential oils, and exogenous enzymes [1],[2]. Consequently, several feed additives have been developed for animal production to modify rumen fermentation and improve animal productivity [2],[3].

Studies have indicated that feeding tree leaves to ruminants can reduce enteric CH_4_ emissions and serve as a viable alternative protein source for livestock [4],[5]. *Moringa oleifera*, a versatile tree, is used as fodder for ruminants. It is now cultivated across many tropical and subtropical regions and is valued for its adaptability and high nutritional potential. This tree is in countries such as, India, Sri Lanka, Bangladesh, Africa, America, Afghanistan, and West Asia. The Moringa tree can adapt to the dry climatic conditions of semi-arid and arid regions and thrive in humid areas during the summer season. It can be grown in arid regions with limited rainfall and is adaptable to a wide range of soil types. Known as a "miracle tree" due to its versatility and adaptability, *M. oleifera* is rich in nutrients and secondary metabolites with health benefits [2],[6]. *M. oleifera* leaves are an excellent source of nutrients, including total phenols, protein, calcium, potassium, magnesium, iron, manganese, and copper, according to a study by Owon et al. [7]. They are also rich in carotenoids, tocopherols, and ascorbic acid, which are phytonutrients beneficial to animal and human health [8]. Moreover, *M. oleifera* contains high levels of polyphenols, including flavonoids, tannins, and phenolic acids, which contribute to its bioactive properties [2],[7].

In cattle nutrition, the primary role of *Moringa* is as a protein supplement. Studies have indicated that *Moringa* by-products may also have the potential to reduce methanogenesis [9],[10]. *M. oleifera* contains high concentrations of secondary metabolites, such as tannins, saponins, and phenolic compounds [11]. These compounds can suppress methanogens, including *Methanobrevibacter* spp., *Methanomicrobium* spp., *Methanobacterium* spp., *Methanosarcina* spp., and other methanogenic archaea [12]. Saponins can reduce CH_4_ production by inhibiting ruminal protozoa [13]. Tannins can reduce CH_4_ production by up to 50% [14] due to their antimicrobial properties, which inhibit certain ruminal CH_4_-producing bacteria and protozoa by binding to dietary proteins and microbial cell enzymes [15]. Thus, *Moringa* leaves can effectively modify rumen fermentation pathways and suppress methanogen growth [16]. The antimethanogenic effects of *Moringa* are likely due to its ability to disrupt microbial cell membranes and alter hydrogen availability, redirecting fermentation toward propionate production, which competes with methanogenesis.

The use of *Moringa* leaf extract (ML), rather than leaf powder or crude extracts, was chosen for this study because it provides a concentrated source of bioactive compounds, enabling lower inclusion rates in the diet while delivering a consistent and standardized dose. Unlike leaf powder, which may vary in composition due to drying and storage conditions, or crude extracts, which require complex processing and may lose some active compounds, leaf extract maximizes the availability of phytochemicals such as polyphenols, saponins, and glycerol derivatives, enhancing its antimethanogenic potential in ruminant diets. Therefore, our objectives of this study were to evaluate the effects of varying levels of ML supplementation on in vitro nutrient degradability, ruminal fermentation, CH_4_ emissions, and the methanogen community structure using the in vitro gas production (GP) technique. We hypothesized that ML would reduce CH_4_ emissions by suppressing methanogenic archaea and altering rumen fermentation toward more energy-efficient pathways, thereby improving nutrient utilization and supporting sustainable ruminant production.

## 2. Materials and methods

### 2.1. Experimental location and ethical approval

The study was conducted at the Advanced Animal Nutrition Laboratory, Department of Animal and Fish Production, Faculty of Agriculture, Alexandria University, Egypt. Animal care and handling were conducted in accordance with ethical standards of the Institutional Animal Care and Use Committee of Alexandria University (approval number Alex. Agri. 082507310), ensuring compliance with animal welfare guidelines during rumen fluid collection.

### 2.2. Moringa extract and the experimental diets

The ML was obtained from the Egyptian Scientific Society of Moringa (ESSM), National Research Centre, Egypt. It was prepared from mature, air-dried leaves using a cold-press extraction method (ESSM proprietary protocol) and stored in dark glass bottles at 4 °C until use. The ML was lyophilized using a freeze dryer to obtain the dried extract. A 10 mL portion of this extract was subjected to gas chromatography–mass spectrometry (GC–MS) analysis (Thermo Scientific, Trace GC Ultra/ISQ Single Quadrupole MS) equipped with a TG-5MS fused-silica capillary column (30 m length, 0.25 mm internal diameter, 0.1 mm film thickness). Tentative compound identification was carried out by comparing the relative retention times and mass spectra with those in the NIST and WILLY spectral libraries associated with the GC–MS system [17].

Four experimental total mixed rations were formulated for the in vitro assay, each comprising 50% concentrate mixture and 50% roughage (a combination of clover *Trifolium alexandrinum* hay and wheat straw), and supplemented with varying levels of ML. The treatments included: (1) Control—basal diet without ML supplementation; (2) ML1—basal diet supplemented with 1 mL ML/100 g dry matter (DM); (3) ML2—basal diet supplemented with 2 mL ML/100 g DM; and (4) ML3—basal diet supplemented with 3 mL ML/100 g DM. The ML was procured from a commercial supplier and analyzed for its secondary metabolites (tannins, saponins, and phenolic compounds) to ensure consistency. The ingredients of the concentrate mixture, proximate composition of the basal diet on a DM basis, and the estimated nutritional value based on predictive models are detailed in Table 1. Prior to incubation, the basal diet was ground using a Wiley mill to pass through a 1-mm screen, ensuring a uniform particle size for accurate *in vitro* evaluation.

**Table 1.** Ingredients and chemical composition of experimental basal diet.

Ingredients	g/kg DM
Berseem hay	500
Ground yellow corn	345
Soybean meal	150
Mineral and vitamin mixture^1^	5

Chemical composition	g/kg DM

Organic matter	914
Crude protein	141
Ether extract	31
Nonfibrous carbohydrates	182
Neutral detergent fiber	560
Acid detergent fiber	287
Acid detergent lignin	59
Hemicellulose	273
Cellulose	228
Total digestible nutrients	832
Relative feed value	111
Relative feed quality	3.18
Net energy lactation (Mcal/kg)	1.94

^1^Each kg contained: 45.8 g dicalcium phosphate, 15 g magnesium sulfate, 6.15 g ferrous sulfate, 0.393 g potassium iodide, 0.753 g copper sulfate, 0.248 g cobalt sulfate, 0.373 g zinc sulfate, and 0.02 g selenite sodium.

Rumen contents were collected from three adult, fasted, slaughtered Egyptian bulls at the slaughterhouse of the Faculty of Agriculture, Alexandria University. All bulls were of the same local breed, similar in age (4 ± 1 years), and comparable in body weight (420 ± 10 kg). They had been maintained on the same diet (50:50 (DM basis) diet of commercial concentrate and clover hay) prior to slaughter to ensure uniformity of the rumen inoculum. Ruminal liquid and solid fractions were collected separately from each animal immediately post-slaughter and kept in pre-warmed thermos containers (39 °C) under anaerobic conditions. The liquid fraction was obtained using a stainless-steel probe (2.5 mm screen) attached to a large-capacity syringe. Equal volumes (1:1 v/v) of both fractions were blended for 10 s, filtered through three layers of cheesecloth, and maintained in a water bath (39 °C) under CO_2_ until inoculation occurred. This process was completed within 30 minutes to preserve microbial viability. Rumen contents from the three bulls were then pooled to form a single composite inoculum, which was used for all incubations. For each treatment, four bottles were prepared: Two for determining true degradability of DM (TDDM) and organic matter (TDOM) and two for pH, ammonia nitrogen (NH_3_-N), volatile fatty acids (VFAs), and protozoal count analyses. The same approach was used for the blank inoculum (bottle without substrate containing inoculum + medium) to correct for GP from the inoculum. An internal standard (clover hay, used as a reference substrate to monitor inoculum activity and allow comparison with previous studies) was included for each inoculum to enable adjustments among inocula. Inocula with variations above 10% were rejected. Blank and standard bottles were incubated in triplicate to ensure reproducibility. Each treatment was evaluated in two incubation runs, with four replicates per treatment per run.

### 2.3. In vitro gas and methane production

A semi-automatic GP system, consisting of a pressure transducer and a data logger (Pressure Press Data GN200, Sao Paulo, Brazil), was used based on the protocol outlined by Bueno et al. [18]. Samples (500 mg as-fed) of the experimental diets were accurately weighed into 120-mL glass bottles, then incubated with 30 mL of MB9 buffer solution [19] and 15 mL of inoculum, resulting in a headspace of 75 mL. The MB9 buffer was prepared in distilled water to 1 L and consisted of the following (g/L): 2.8 g NaCl, 0.1 g CaCl₂, 0.1 g MgSO₄·7H_2_O, 2.0 g KH_2_PO₄, and 6.0 g Na₂HPO₄. The pH was then adjusted to 6.8 using CO_2_ flushed for 15 min. The bottles were sealed immediately with 20 mm butyl rubber septum stoppers (Bellco Glass Inc., Vineland, NJ, USA), manually mixed, and incubated at 39 °C in a forced-air oven for 24 h. Headspace gas pressure was measured after 3, 6, 9, 12, and 24 h. Gas production was calculated using the equation: V = 4.974 × p + 0.171 (n = 500; r² = 0.98; data not reported), where V=gas volume (mL) and p=measured pressure (psi).

For CH_4_ analysis, gas was collected from the bottles after 3, 6, 9, 12, and 24 h incubation using a (2 mL) syringe each time and accumulated in vacutainer tubes. Methane was determined by an Agilent 7890 gas chromatograph with a three-valve system using 1/8-inch packed columns with early back flush of the C6 components, equipped with a thermal conductivity detector. Separation was achieved using a micro-packed column; helium was the carrier gas with a flow rate of 28.0 mL/min. The detector and column temperatures were 250 °C and 60 °C, respectively. The system was calibrated using a standard gas curve in the range of expected sample concentrations. The calibration ensured a detection limit of 0.1% for accurate CH_4_ quantification. Methane production at the end of incubation was calculated as described by Tavendale et al. [20]: CH_4_, mL = (total gas volume + headspace) × CH_4_ concentration, mL/mL. GP and CH_4_ were expressed as mL/g DM and mL/g TDOM after correcting the values of total GP and incubated or TDOM for the corresponding blank.

### 2.4. Rumen degradability and fermentation characteristics

After terminating incubation at 24 h, TDDM and TDOM were determined in two bottles for each inoculum by adding 50 mL of neutral detergent solution [21] and incubating at 105 °C for 3 h. The bottle contents were filtered into pre-weighed crucibles, washed with hot water, then with acetone, and the residual DM and ash were determined thereafter. The partitioning factor (PF) was calculated as the ratio of mg of TDOM to gas volume (mL) at 24 h incubation [22]. The contents of the other two bottles were used for determining fermentation characteristics. The content of each bottle was transferred to a centrifuge tube and centrifuged at 3000 rpm for 15 min. Five mL of the supernatant was transferred to a 10 mL glass bottle and stored at −20 °C until they were analyzed for NH_3_-N and VFA determination. Fermentation pH was recorded using a digital pH meter (GLP 21, CRISON, Barcelona, Spain). Protozoal counts were assessed using a Digital Zoom Video Microscope (LCD 3D, GiPPON, Hong Kong) following Dehority et al. [23]. Volatile fatty acids (VFAs) were quantified by gas chromatography (TRACE1300, Thermo Fisher Scientific, Milan, Italy) with an autosampler (AS3800) and HP-FFAP capillary column (19091F-112; Agilent Technologies, Palo Alto, CA, USA) using standards from Sigma Chemie GmbH (Steinheim, Germany), as per Palmquist and Conrad [24]. VFA samples were centrifuged at 10,000 × g for 10 minutes at 4 °C and filtered through a 0.22-µm membrane to remove particulates before analysis using gas chromatography (GC). Rumen NH_3_-N was determined colorimetrically using a commercial kit (Biodiagnostic, Giza, Egypt), based on the method of Konitzer and Voigt [25].

### 2.5. Structure of the methanogenic community

#### 2.5.1. DNA extraction from rumen samples

Total DNA was extracted from rumen contents using the cetyltrimethylammonium bromide (CTAB) and the phenol–chloroform–isoamyl alcohol method. A total of 500 µL of prewarmed DNA extraction buffer (1.5 mol/L Tris, pH 7.6; 0.4 mol/L disodium salt of ethylenediaminetetraacetic acid (Na_2_EDTA; Merck, Darmstadt, Germany); 2.5 mol/L NaCl; 2% cetyltrimethylammonium bromide (CTAB; Merck, Germany)) was diluted to 850 mL with H_2_O. The pH was adjusted to 8.0, and the final volume was adjusted to 1 L, which was added to 500 µL of each sample, followed by 30 µL of 10% SDS (10% sodium dodecyl sulfate (SDS), β-mercaptoethanol, chloroform:isoamyl alcohol (24:1), isopropanol, 70% and 90% ethanol) and mixed gently. This was then incubated at 65 °C for 1 h. After incubation, the mixture was centrifuged at 12,000 rpm for 12 min at 4 °C, and the supernatant was transferred to a fresh, sterilized centrifuge tube, and then an equal volume of phenol, chloroform, and isoamyl alcohol (25:24:1) was added and mixed well. This was then centrifuged at 12,000 rpm for 12 min at 4 °C. To a fresh, sterilized tube, an equal volume of chloroform was added to the supernatant, and the mixture was centrifuged at 12,000 rpm for 12 min at 4 °C; this step was repeated once more. The supernatant was transferred to a fresh, sterilized tube, and 1 mL of absolute ethanol was added. The tube was then mixed and kept at −20 °C overnight. The sample was centrifuged at 12,000 rpm for 15 min at 4 °C, the supernatant was discarded without disturbing the pellet, and 1 mL of 70% ethanol was added. This was then centrifuged at 14,000 rpm for 15 min at 4 °C, the supernatant was discarded, and the pellet was air-dried at 37 °C. The pellet was resuspended in 30 µL of DEPC-treated H_2_O, and the DNA solution was stored at −20 °C for future use. The genomic DNA concentration was measured using a NanoDrop spectrophotometer (Bio-Rad, USA). DNA integrity was verified by running 5 µL of each sample on a 1% agarose gel to confirm the absence of degradation.

#### 2.5.2. PCR amplification from the purified rumen DNA

PCR amplification was performed using an Agilent 8800 Thermocycler (Applied Biosystems, USA) with methanogen-specific forward and reverse primers. Methanogenic 16S rDNA genes were amplified from each DNA sample. A nested PCR program was used to amplify the partial 16S rRNA gene for enhanced detection of methanogens. A primary PCR was conducted using the universal primer pair Met86f (5′-GCTCAGTAACACGTGG-3′) [26] and Met915r (5′-GTGCTCCCCCGCCAATTCCT-3′) [27] following the conditions described by Zhou et al. [28]. PCR amplification was carried out on DNA from individuals and was performed in a total reaction mixture volume of 20 µL containing: 10 µL of PCR Master Mix 2X, 2 µL of 10 µM primer, 1 µL of DNA (50 ng), and 7 µL of nuclease-free H_2_O. The PCR amplification program started with initial denaturation at 95 °C for 3 min, followed by 35 cycles: Denaturation at 95 °C for 45 s, annealing at 55.5 °C for 45 s, extension at 72 °C for 1 min 40 s, with a final extension at 72 °C for 5 min. PCR products were separated on a 1.5% agarose gel and stained with ethidium bromide (Sigma, Germany), visualized on a UV transilluminator, and photographed by a Gel Documentation System (Gel Doc AlphaChem Imager, USA). Negative controls (no template DNA) were included to rule out contamination.

Subsequently, 1 µL of PCR product was used as a template for the second amplification with the forward primer uniMet1-F (5′-CCGGAGATGGAACCTGAGAC-3′) and the reverse primer uniMet1-R (5′-CGGTCTTGCCCAGCTCTTATTC-3′) using the following conditions: initial denaturation for 3 min at 94 °C, followed by 35 cycles with denaturation for 30 s at 94 °C, annealing for 30 s at 57.5 °C, and extension for 1 min at 72 °C with a final extension at 72 °C for 5 min. The specificity of these primers ensured amplification of methanogen-specific sequences, minimizing non-target amplification. This primer pair targets a segment within the V3–V4 region of the archaeal 16S rRNA gene, a region known for providing strong phylogenetic resolution among methanogenic taxa. The uniMet1 primer set has been validated in previous studies for its high specificity toward major methanogenic lineages, including Methanobrevibacter, Methanosphaera, Methanobacterium, and Methanosarcina, ensuring accurate representation of methanogen community composition.

#### 2.5.3. DNA sequencing for the nested PCR products

The PCR amplicons (~179 bp) were subjected to PCR purification using a Qiagen PCR Purification Kit, and the purified PCR product was submitted to Macrogen Company for sequencing (Macrogen, Korea). Purification removed residual primers and dNTPs, ensuring high-quality sequencing data. Since only four short (~179 bp) amplicons were sequenced, this method offers qualitative information about the phylogenetic placement of a small number of methanogen sequences rather than a quantitative assessment of the community. PCR on its own cannot characterize overall methanogen composition or diversity, so any differences among treatments are interpreted as shifts in the phylogenetic affiliation of these few sequences rather than true changes in community structure.

#### 2.5.4. Sequence analysis and phylogenetic investigation

Sanger sequencing generated individual consensus sequences for each amplicon. The DNA BLAST was performed to assess nucleotide identity compared with sequences in GenBank (https://blast.ncbi.nlm.nih.gov/Blast.cgi). Sequence alignment was performed using the ClustalW program (https://www.genome.jp/tools-bin/clustalw). The phylogenetic tree was constructed using the MEGA11 program (https://www.megasoftware.net/). Phylogenetic trees were constructed using the neighbor-joining method with 1,000 bootstrap replicates to ensure robust clustering of methanogen taxa.

### 2.6. Statistical analysis

Data were analyzed by one-way ANOVA using the general linear model procedure (PROC GLM) of SAS 9.4 (SAS Inst., Inc., Cary, NC). The results were subjected to analysis of variance using the following statistical model: Y_ij_ = µ + T_i_ + e_ij_, where Y_ij_ = observed value, µ = overall mean, T_i_ = treatment effect, and e_ij_ = residual error. Differences among treatments were considered significant at (P < 0.05), and trends were noted if (P < 0.10). Tukey's test for multiple comparisons was used to detect differences among treatment means. Linear and quadratic contrasts were performed to evaluate dose-response effects of ML supplementation, and data normality was verified using the Shapiro-Wilk test.

## 3. Results

The GC-MS analysis of ML revealed a diverse chemical composition comprising various low- and high-molecular-weight compounds (Table 2). Among the identified compounds, glycerin (1,2,3-propanetriol) was the predominant constituent, accounting for 82.08% of the total chromatographic area. In addition to glycerin, several unsaturated fatty acid derivatives were detected at retention time 29.58 minutes, each contributing 4.74% to the total area. These include 9,12-octadecadienoic acid (Z,Z) (commonly known as linoleic acid), 9,12-octadecadienoyl chloride (Z,Z), and 17-octadecynoic acid. Minor yet bioactive compounds were also present in the extract, including sulfur- and nitrogen-containing metabolites such as L-cystathionine and DL-homocysteine, S-ethyl-, each detected in concentrations ranging from 0.69% to 1.54%. Uridine 5′-diphosphate was also identified in similar concentrations. Other compounds, such as 1,3-dicyclohexylurea and tetramethylphosphonium cation, were detected at concentrations below 2%.

**Table 2.** Principal identified phytoconstituents of *Moringa oleifera* leaf extract analyzed by gas chromatography–mass spectrometry.

RT (min)	Compound Name	Concentration (%)^1^	Molecular Formula	MW	CAS Number
11.40	1,3-Dicyclohexylurea	0.69	C_13_H_24_N_2_O	224	2387-23-7
11.40	Tetramethylphosphonium cation	0.69	C_4_H_12_P	91	32589-80-3
11.40	Uridine 5′-diphosphate	0.69	C_9_H_14_N_2_O_12_P_2_	404	58-98-0
11.40	DL-Homocysteine, S-ethyl-	0.69	C_6_H_13_NO_2_S	163	67-21-0
11.40	L-Cystathionine	0.69	C_7_H_14_N_2_O_4_S	222	56-88-2
11.68	Glycerin (1,2,3-Propanetriol)	0.79	C_3_H_8_O_3_	92	56-81-5
12.06	1,3-Dicyclohexylurea	1.53	C_13_H_24_N_2_O	224	2387-23-7
12.14	L-Cystathionine	1.23	C_7_H_14_N_2_O_4_S	222	56-88-2
12.58	Glycerin (1,2,3-Propanetriol)	1.19	C_3_H_8_O_3_	92	56-81-5
12.82	Glycerin (1,2,3-Propanetriol)	1.94	C_3_H_8_O_3_	92	56-81-5
13.13	Glycerin (1,2,3-Propanetriol)	1.54	C_3_H_8_O_3_	92	56-81-5
13.54	Glycerin (1,2,3-Propanetriol)	82.08	C_3_H_8_O_3_	92	56-81-5
29.58	9,12-Octadecadienoyl chloride (Z,Z)	4.74	C_18_H_31_ClO	298	7459-33-8
29.58	9,12-Octadecadienoic acid (Z,Z)	4.74	C_18_H_32_O_2_	280	60-33-3
29.58	17-Octadecynoic acid	4.74	C_18_H_32_O_2_	280	34450-18-5

RT = retention time (min); MW = molecular weight of the compound (g/mol). ^1^Concentration based on the total areas of the identified peaks.

The effects of ML supplementation on ruminal net GP, TDDM, TDOM, and PF after 24 h in vitro incubation are presented in Table 3. GP (linear, P < 0.001; quadratic, P = 0.002) and CH_4_ (linear, P = 0.033) decreased with supplementation of all ML levels compared to the control group. Specifically, net GP decreased from 179.2 mL/g DM in the control to 163.9, 160.8, and 159.8 mL/g DM for ML1, ML2, and ML3, respectively, representing reductions of 8.3%, 10.0%, and 10.7%. Similarly, CH_4_ production declined from 30.8 mL/g DM in the control to 26.6 mL/g DM (ML1), 26.5 mL/g DM (ML2), and 26.6 mL/g DM (ML3), corresponding to a 13.6–13.9% reduction.

Similarly, TDDM (linear, P = 0.038) and TDOM (linear, P = 0.016) decreased. TDDM decreased from 748.6 g/kg DM in the control to 677.3 g/kg DM in ML1, 696.8 g/kg DM in ML2, and 681.2 g/kg DM in ML3 (linear, P = 0.038), while TDOM decreased from 755.5 g/kg DM to 688.9, 708.6, and 691.0 g/kg DM for ML1, ML2, and ML3, respectively. In contrast, PF increased (quadratic, P = 0.002) with supplementation of both ML1 and ML2 levels reaching 4.33 mg TDOM/mL GP for ML1 and 4.30 mg TDOM/mL GP for ML2 compared with 3.97 mg TDOM/mL GP in the control group.

**Table 3.** Effects of Moringa leaf extract on ruminal net GP, TDDM, TDOM, and PF for 24 h *in vitro*.

	Treatments^1^				SEM	P value		
Items	Control	ML1	ML2	ML3		Treatment	Linear	Quadratic
GP (mL/g DM)	179.2^a^	163.9^b^	160.8^b^	159.8^b^	1.76	0.001	<0.001	0.002
CH_4_ (mL/g DM)	30.8^a^	26.6^b^	26.5^b^	26.6^b^	0.99	0.006	0.033	0.087
TDDM (g/kg DM)	748.6^a^	677.3^b^	696.8^ab^	681.2^b^	18.19	0.046	0.038	0.145
TDOM (g/kg DM)	755.47^a^	688.9^b^	708.6^b^	691.0^b^	14.58	0.017	0.016	0.112
PF, mg TDOM/mL GP	3.97^b^	4.33^a^	4.30^a^	3.86^b^	0.090	0.003	0.506	0.002

GP (mL/g DM) = net gas production per gram of dry matter; CH_4_ (mL/g DM) = methane production per gram of dry matter; TDDM (g/kg DM) = truly degraded dry matter; TDOM (g/kg DM) = truly degraded organic matter; PF (mg TDOM/mL GP) = partitioning factor, calculated as milligrams of truly degraded organic matter per milliliter of gas produced. Different superscript letters within the same row denote significant differences (P < 0.05); SEM = standard error of the mean. ^1^Treatments consisted of a control group fed a basal diet without additives (50% concentrate + 50% forage), and three groups supplemented with ML at levels of 1.0, 2.0, and 3.0 mL per 100 g DM, designated as ML1, ML2, and ML3, respectively.

The effects of ML supplementation on rumen microbial fermentation parameters after 24 h of incubation in vitro are shown in Table 4. Treatments did not affect ruminal pH (5.41–5.47) or NH_3_-N (17.2–19.6 mg/dL). All ML levels decreased total VFA, with concentrations dropping from 109.2 mM in the control to 102.9 mM (ML1), 97.5 mM (ML2), and 105.2 mM (ML3) (linear, P = 0.009; quadratic, P = 0.003). Butyrate was particularly sensitive to ML, decreasing from 14.17 mM in the control to 11.8 mM (ML1), 10.1 mM (ML2), and 7.6 mM (ML3) (linear, P < 0.001). Acetate and propionate concentrations also declined with moderate ML inclusion, with acetate decreasing from 65.2 mM (control) to 60.8 mM (ML1) and 59.9 mM (ML2), and propionate from 15.6 mM (control) to 14.6 mM (ML1) and 14.4 mM (ML2) (quadratic, P = 0.005). Conversely, branched-chain VFA increased with high ML supplementation. Isobutyrate rose from 10.3 mM in the control to 13.6 mM in ML3 (linear, P = 0.004; quadratic, P = 0.012) and isovalerate increased from 2.7 mM to 3.3 mM (linear, P = 0.023; quadratic, P = 0.012).

**Table 4.** Effects of Moringa leaf extract on rumen microbial fermentation parameters for 24 h incubation *in vitro*.

	Treatments^1^				SEM	P value		
Items	Control	ML1	ML2	ML3		Treatment	Linear	Quadratic
pH	5.44	5.42	5.47	5.41	0.049	0.787	0.828	0.693
Ammonia-N, mg/dL	17.2	18.6	19.6	18.8	0.85	0.256	0.132	0.204
Volatile fatty acids, mM		
Total	109.2^a^	102.9^b^	97.5^c^	105.2^b^	1.10	0.001	0.009	0.003
Acetate	65.2^a^	60.8^bc^	59.9^c^	64.1^ab^	1.06	0.027	0.421	0.005
Propionate	15.6^a^	14.6^bc^	14.4^c^	15.4^ab^	0.25	0.025	0.421	0.005
Butyrate	14.17^a^	11.8^b^	10.1^c^	7.6^d^	0.46	0.001	<0.001	0.943
Isobutyrate	10.30^bc^	11.4^b^	9.6^c^	13.6^a^	0.42	0.001	0.004	0.012
Valerate	1.27	1.40	1.16	1.16	0.060	0.099	0.071	0.236
Isovalerate	2.70^b^	2.77^b^	2.43^b^	3.30^a^	0.111	0.005	0.023	0.012

Different superscript letters within the same row denote significant differences (P < 0.05); SEM = standard error of the mean. ^1^Treatments consisted of a control group fed a basal diet without additives (50% concentrate + 50% forage), and three groups supplemented with ML at levels of 1.0, 2.0, and 3.0 mL per 100 g DM, designated as ML1, ML2, and ML3, respectively.

The effects of ML supplementation on ruminal protozoal counts are shown in Table 5. Total protozoal counts were not significantly affected by ML supplementation, ranging from 6.73 × 10^5^/mL (ML3) to 7.87 × 10^5^/mL (control). Supplementation with ML levels decreased (linear, P = 0.008) *Diplodinium* spp. Counts from 1.30 × 10^5^/mL in the control to 0.95 × 10^5^/mL (ML1), 0.93 × 10^5^/mL (ML2), and 0.83 × 10^5^/mL (ML3). Additionally, ML1 decreased (quadratic, P=0.048) Epidinium spp. counts compared to the control group (0.03 × 10^5^/mL vs 0.33 × 10⁵/mL). The remaining protozoal genera, including Entodinium, Eudiplodinium, Isotrica, and Ophryscolex, were largely unaffected.

**Table 5.** Effects of Moringa leaf extract levels supplementation on protozoal count (10^5^/mL) in in vitro experiment.

	Treatments^1^				SEM	P value		
Items	Control	ML1	ML2	ML3		Treatment	Linear	Quadratic
Total	7.87	6.99	7.52	6.73	0.587	0.544	0.304	0.930
*Entodinium*	5.77	5.87	5.88	5.43	0.472	0.909	0.666	0.587
*Diplodinium*	1.30^a^	0.95^b^	0.93^b^	0.83^b^	0.100	0.030	0.008	0.177
*Epidinium*	0.33^a^	0.03^b^	0.27^a^	0.20^ab^	0.062	0.017	0.357	0.048
*Eudiplodinium*	0.32	0.10	0.35	0.15	0.081	0.093	0.593	0.894
*Isotrica*	0.13	0.02	0.07	0.10	0.043	0.313	1.000	0.144
*Ophryscolex*	0.017	0.017	0.017	0.017	0.000	1.000	1.000	1.000

Different superscript letters within the same row denote significant differences (P < 0.05); SEM = standard error of the mean. ^1^Treatments consisted of a control group fed a basal diet without additives (50% concentrate + 50% forage), and three groups supplemented with ML at levels of 1.0, 2.0, and 3.0 mL per 100 g DM, designated as ML1, ML2, and ML3, respectively.

**Figure 1.**
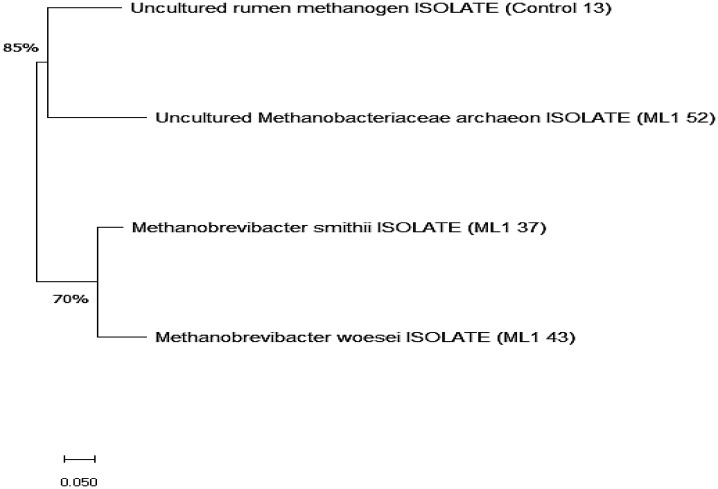
Phylogenetic analysis of methanogen sequences in the rumen.

The phylogenetic tree reveals two separated major clades within the methanogenic community (Figure 1). The first, supported by a relatively high bootstrap value of 85%, clusters the uncultured rumen methanogen isolate (Control 13) with the uncultured *Methanobacteriaceae archaeon* isolate (ML1 52), indicating a shared recent ancestor and suggesting that these two taxa belong to a closely related subgroup within the Methanobacteriaceae. Their tight clustering reflects strong genetic similarity, consistent with uncultured rumen-associated methanogens that often display limited divergence. The second major clade, supported by a 70% bootstrap value, contains *Methanobrevibacter smithii* (ML1 37) and *Methanobrevibacter woesei* (ML1 43). These two isolates form a robust lineage typical of the genus Methanobrevibacter, reflecting well-established evolutionary separation from the uncultured Methanobacteriaceae lineage above. Their grouping suggests that they share a more recent common ancestor with each other than with any of the uncultured isolates.

**Figure 2.**
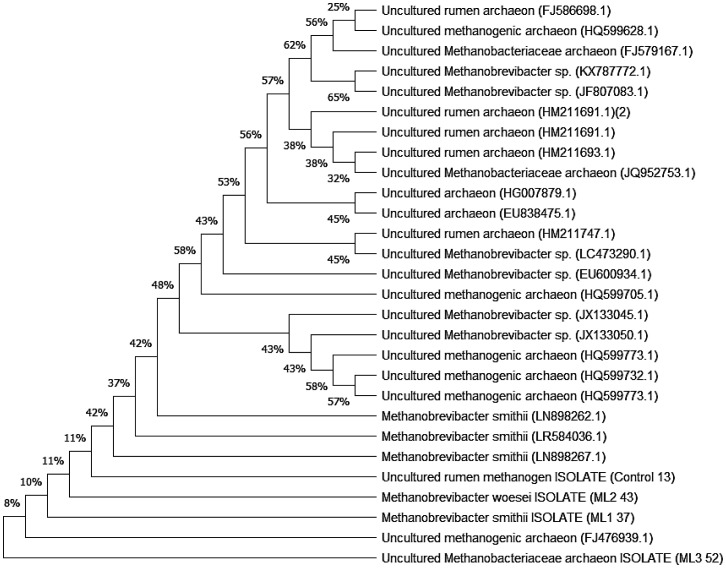
Comparative Phylogenetic Analysis of Four Study Isolates of methanogens and Gene Bank Data

The phylogenetic analysis reveals a broad and heterogeneous methanogenic community in which most sequences cluster within uncultured rumen-associated archaeal lineages (Figure 2). The upper two-thirds of the tree are dominated by highly diverse uncultured Methanobacteriaceae and Methanobrevibacter-like groups, reflecting the well-known taxonomic complexity of rumen methanogens. Many of these environmental sequences branch with moderate bootstrap support (32–65%), indicating substantial genetic diversity and suggesting that multiple distinct but related ancestral lineages coexist within the rumen environment.

Within this large environmental cluster, several strongly supported subclades are formed by uncultured *M. archaeon* and uncultured Methanobrevibacter sequences (e.g., clusters supported at 56%, 57%, 62%, and 65%). These groups represent evolutionary lineages that retain conserved 16S rRNA gene motifs typical of rumen methanogenic archaea but have not been taxonomically resolved due to the absence of cultured representatives. Toward the lower part of the tree, the phylogeny becomes more resolved, with a distinct and well-defined clade consisting of the reference strains *M. smithii* (LN898262.1, LRS84036.1, LN898267.1) and isolates ML1 37 and ML2 43. Their close grouping indicates a strong evolutionary relationship and confirms that these isolates belong firmly within the *M. smithii/woesei* lineage. The bootstrap values associated with this cluster (57–58%) demonstrate moderate but consistent support for their shared ancestry.

Just below this clade, the isolate ML3 52 forms a separate branch together with an uncultured *M. archaeon* (JF476939.1), suggesting that ML3 52 represents a lineage more closely related to ancestral Methanobacteriaceae rather than to the more derived *M. smithii* group. In contrast, Control 13 branches near the base of the *Methanobrevibacter subclade*, clustering with uncultured rumen methanogen sequences at the lower part of the tree. This position indicates that the Control 13 isolate shares ancestry with a distinct environmental methanogen lineage but remains evolutionarily separate from the *M. smithii* group and the Methanobacteriaceae associated ML3 52.

## 4. Discussion

The chemical profile of ML, as revealed by GC-MS analysis, highlights its potential as a functional feed additive in ruminant nutrition. The predominance of glycerin (1,2,3-propanetriol), which comprised over 82% of the total identified compounds, positions ML as an excellent alternative energy source in animal diets. Glycerin is a glucogenic compound that bypasses rumen fermentation to a large extent and is absorbed in the small intestine or converted into propionate in the rumen [29]. Its supplementation has been associated with improved energy availability, especially in early-lactation dairy cows, and can help prevent negative energy balance [30]. Importantly, glycerin has been shown to reduce enteric CH_4_ production, typically by increasing propionate formation and thereby decreasing the acetate:propionate ratio in the rumen, as propionate formation competes with methanogenesis for hydrogen utilization [29]. In the present study, ML1 and ML2 reduced both propionate and CH_4_, which appears contrary to this mechanism. This discrepancy may reflect the complex interactions of other bioactive compounds in ML, such as polyphenols and fatty acids, which can inhibit methanogens directly or shift overall fermentation patterns, reducing CH_4_ independently of propionate formation [31],[32]. Therefore, the CH_4_-lowering effect observed with ML1 and ML2 likely involves multiple mechanisms beyond glycerin-induced glucogenic effects, highlighting the need for further mechanistic studies. Another notable group of compounds identified includes unsaturated fatty acids, particularly 9,12-octadecadienoic acid (Z,Z) (linoleic acid), and its derivative linoleoyl chloride, each accounting for about 4.74% of the extract. Linoleic acid is a bioactive compound with well-documented effects on rumen fermentation and microbial populations. Upon biohydrogenation in the rumen, linoleic acid leads to the production of conjugated linoleic acid (CLA), which has health benefits for animals and consumers. From a rumen fermentation standpoint, unsaturated fatty acids exert toxic effects on certain methanogens and rumen protozoa, thereby contributing to lower CH_4_ emissions [33]. Additionally, these fatty acids can suppress protozoal populations, indirectly reducing the numbers of symbiotic methanogens that reside within protozoa [33].

The presence of sulfur-containing amino acid derivatives, such as DL-homocysteine, S-ethyl-, and L-cystathionine, though present in relatively small concentrations (0.69–1.54%), may have a modulatory effect on microbial protein synthesis and antioxidant status. Sulfur amino acids are critical for microbial growth and are precursors for key molecules like glutathione, which play a role in maintaining microbial redox balance. These compounds may also support microbial efficiency and stabilize rumen fermentation, especially under oxidative or dietary stress.

The identification of uridine 5′-diphosphate, a nucleotide sugar involved in the synthesis of glycoproteins and glycolipids, may suggest a role in enhancing gut barrier integrity and immune modulation [34]. While this compound's direct effect on rumen fermentation is not well established, its presence in feed could potentially support epithelial cell function and immune resilience, especially in young or stressed animals.

Overall, the composition of ML suggests that it may have dual functionality in ruminant feeding systems: (1) as an energy-dense additive due to its high glycerol content, and (2) as a CH_4_ mitigation strategy through the antimicrobial actions of its unsaturated fatty acids. Its inclusion in ruminant diets may shift fermentation pathways toward more propionate production, reduce protozoal and methanogenic activity, and ultimately improve feed efficiency [35]. The bioactivity of ML is especially relevant in the context of climate-smart livestock feeding strategies, where reducing CH_4_ emissions, enhancing nutrient utilization, and stabilizing the rumen microbial ecosystem are top priorities. In future in vivo studies, researchers should aim to quantify the effects of ML inclusion on feed intake, digestibility, VFA profiles, microbial protein yield, and CH_4_ output, while monitoring shifts in rumen bacterial and protozoal populations using molecular tools.

Secondary metabolites in ML, such as tannins, flavonoids, phenolics, and saponins, can enhance nutrient digestibility in ruminants. These metabolites can be utilized as energy sources by rumen microbes without negatively affecting rumen fermentation. Moreover, they possess antibacterial and antiprotozoal properties that can reduce CH_4_ production while increasing acetate production, thereby enhancing carbohydrate digestion in ruminants. Additionally, they influence ruminal cellulolytic and ammonia-producing bacteria, limiting the production of gases required for methanogenesis [12]. The presence of α-linolenic acid in ML may further contribute to CH_4_ reduction by altering microbial membrane fluidity, inhibiting methanogen activity [32].

Animal feed composition plays a critical role in controlling CH_4_ emissions. In recent years, approaches to reduce CH_4_ emissions from ruminants have involved adding specific inhibitors to their feed. These inhibitors can be chemical, biological, or natural and work by suppressing the growth of methanogenic microbes in the rumen. One effective natural inhibitor is *M. oleifera* leaves, which can modify rumen fermentation pathways [36]. The present study revealed that the addition of ML significantly reduced CH_4_ production. This reduction is likely due to the presence of α-linolenic acid, tannins, and saponins in ML [32]. The presence of tannins and phenolic compounds exerted antimicrobial effects, which were a primary cause of CH_4_ reduction [14]. A reduction in CH_4_ was also observed in ML-treated ruminants compared to soybean meal [9]. Supplementation of ML by replacing soybean meal significantly reduced CH_4_ production and ammonia-N, but increased CO_2_ production [9]. ML feeding decreased enteric CH_4_ emissions and increased milk production in dairy cows, as reported by Bashar et al. [37]. ML supplementation may reduce energy losses, including CH_4_ and urinary nitrogen, without adversely affecting beef cattle production [38]. However, the increased CO_2_ production observed in some studies suggests a trade-off in greenhouse gas emissions that requires further investigation to optimize ML's environmental benefits.

Kholif et al. [10] showed that the chemical composition of incubated substrates affected in vitro production of CH_4_ and CO_2_ due to its impact on nutrient availability and microbial activity in the rumen. Secondary metabolites possess antimicrobial and antiprotozoal properties, which reduce CH_4_ production [12]. Additionally, secondary metabolites affect ruminal cellulolytic bacteria [13] and reduce the availability of gases required for methanogenesis (i.e., CO_2_ and H_2_) [14]. Kholif & Olafadehan [12] noted that secondary metabolites in plants inhibit ruminal CH_4_-producing bacteria and decrease the availability of H_2_ for methanogenesis. Goel and Makkar [14] observed a 50% reduction in CH_4_ production as a result of tannin and phenolic compound administration. The presence of α-linolenic acid in ML may be another reason for decreased CH_4_ production [32]. Similar results were observed by Morsy et al. [9] when replacing soybean meal with *M. oleifera* leaves. It was expected that increasing ML levels in the diet would decrease ruminal bacterial counts due to the antimicrobial effects of secondary metabolites, but this was not observed in this experiment because diets containing ML increased total ruminal bacterial counts. These results confirm that secondary metabolites in ML are within acceptable ranges for ruminal bacterial activity. In their review, Kholif and Olafadehan [12] reported the high capacity of ruminal microbiota to utilize ML metabolites as energy sources. The decreased ruminal protozoa with diets containing ML may partially explain the increase in ruminal bacterial counts. Extracts from leaves of various plants with high flavonoid and tannin levels reduced CH_4_ emissions and increased microbial counts [39]. The increased bacterial counts may enhance fiber degradation in low- ML treatments, but higher doses could inhibit cellulolytic bacteria, as suggested by the reduced TDDM and TDOM.

Other studies have asserted that ML supplementation in the diet of dairy cows not only improved milk yield and milk quality but also regulated microbial metabolic function and CH_4_ emissions [40]. In *in vitro* experiments, rumen emissions were significantly reduced when leaves or extracts of *Moringa* were used, through active modulation of the rumen microbiome [32]. The reduction in protozoal counts, particularly *Diplodinium* and *Epidinium* spp., aligns with the observed decrease in CH_4_, as protozoa harbor methanogens symbiotically.

However, the reduced TDDM and TDOM observed with higher ML levels may inhibit fiber degradation, likely due to tannins binding to dietary fiber, and the presence of other phenolic compounds in ML may limit cellulolytic bacterial activity, which are responsible for degrading cellulose and hemicellulose in the diet. Future research should explore optimal ML dosages to balance CH_4_ mitigation with nutrient digestibility, potentially combining ML with prebiotics or enzymes to enhance fiber degradation. Tannins can bind to dietary fiber and microbial enzymes, forming complexes that reduce substrate accessibility for cellulolytic bacteria, thereby limiting fiber degradation [12],[13],[39]. While moderate levels of ML provide bioactive compounds that support rumen fermentation and reduce CH_4_ emissions, higher concentrations may create a trade-off, where CH_4_ mitigation comes at the cost of reduced fiber and OM degradability. This highlights the need to optimize inclusion rates of ML in ruminant diets to balance environmental benefits with nutrient utilization.

Additionally, the reduced TDDM and TDOM may reflect interactions between tannins and other secondary metabolites, such as saponins and polyphenols, which can further modulate microbial populations, particularly cellulolytic and fibrolytic bacteria. Incorporating complementary strategies, such as exogenous fibrolytic enzymes or prebiotics, may help counteract these inhibitory effects while maintaining CH_4_ mitigation. Additionally, in vivo studies are needed to validate these in vitro findings and assess ML's long-term effects on animal performance and greenhouse gas emissions.

ML supplementation significantly influenced the in vitro rumen fermentation patterns in a dose-dependent manner. Ruminal pH remained stable across all treatments (5.41–5.47), indicating that ML inclusion did not compromise the overall rumen environment required for optimal microbial activity [41]. From a practical perspective, maintaining pH stability while improving degradability is an ideal combination; it implies a fermentation process that is active and balanced. Total VFA concentrations decreased with ML1 and ML2, primarily due to reductions in acetate, propionate, and butyrate, indicating partial suppression of carbohydrate fermentation, likely mediated by bioactive secondary metabolites such as tannins and polyphenols [12]. In contrast, ML3 increased isobutyrate and isovalerate levels, reflecting enhanced deamination of branched-chain amino acids (valine, leucine, and isoleucine) in the rumen. These branched-chain VFAs serve as key substrates for cellulolytic and proteolytic bacteria, supporting microbial protein synthesis [42]. Elevated branched-chain VFA concentrations suggest that higher ML inclusion may promote protein fermentation or microbial turnover, which could partly explain the modest reductions in TDDM and TDOM observed with ML3 [32]. This shift underscores a complex interplay between ML bioactive compounds, amino acid metabolism, and rumen microbial activity. The overall changes in VFA profiles and nutrient degradability highlight a dose-dependent interaction between ML compounds and rumen microbes. Moderate ML levels (ML1 and ML2) reduced total VFA production and butyrate, partly lowering TDDM and TDOM through inhibitory effects on fibrolytic bacteria. Higher supplementation (ML3) redirected fermentation toward amino acid catabolism without substantially compromising overall nutrient utilization. These patterns coincide with observed reductions in CH_4_ production, suggesting that ML's antimethanogenic effects result from multiple mechanisms, including methanogen inhibition, protozoal suppression, and modulation of fermentation pathways toward increased propionate and branched-chain VFA formation.

The decrease in ruminal protozoal counts with ML may be attributed to the secondary metabolites in ML. The presence of saponins and other plant secondary metabolites has been reported to reduce methanogenic archaea and protozoa in the rumen [12],[13]. Tannins have a strong defaunating effect, but the mechanism of action is not fully understood [15]. As noted, the reduced protozoal counts contributed to decreased CH_4_ production. Saponins likely disrupt protozoal cell membranes, reducing their populations and limiting methanogen symbiosis, which aligns with the observed shifts in methanogen community structure [13].

Plant secondary metabolites, like polyphenols and carotenoids, have demonstrated antimicrobial activity (against gram-positive bacteria) and disruption of redox reactions [12]. The effect of ML on rumen microbiota enhances rumen fermentation kinetics in a beneficial manner, leading to improved feed degradability while reducing CH_4_ production, which benefits both the host and the environment [9],[32]. In this study, the sequences of *M. smithii* and *M. woesei* were associated with treatments that reduced CH_4_ production compared to the control group, which was linked to an uncultured *M. archaeon*. Fatty acids and secondary metabolites in ML may mediate significant shifts in microbial populations, as observed here. The significant decrease in protozoa was closely associated with CH_4_ reduction in this study. Secondary metabolites in ML, such as tannins, flavonoids, phenolics, and saponins, may improve nutrient digestibility. Rumen microbes can utilize these compounds as energy sources without negatively impacting rumen fermentation [43]. Moreover, these metabolites have antibacterial and antiprotozoal effects, which reduce CH_4_ production while increasing acetate production, thereby enhancing carbohydrate digestion in ruminants. Additionally, they influence ruminal cellulolytic and ammonia-producing bacteria, reducing the production of gases essential for methanogenesis [12],[14]. The shift in methanogen community structure, with reduced abundance of *Methanobrevibacter* spp. in ML treatments, suggests selective inhibition by ML's bioactive compounds, potentially reshaping rumen microbial ecology over time.

Beyond the observed in vitro effects on rumen fermentation, nutrient degradability, and CH_4_ mitigation, the practical application of ML in real feeding systems warrants careful consideration. Palatability and feed intake are critical factors in vivo, as high concentrations of ML or its associated secondary metabolites could reduce voluntary feed consumption in ruminants. Additionally, the cost and availability of ML may limit its widespread adoption, particularly in large-scale livestock operations, and extraction or processing methods may affect both the concentration of bioactive compounds and overall economic feasibility. Long-term effects of ML supplementation remain uncertain, including potential impacts on animal performance, milk composition, or rumen microbial stability over extended feeding periods. While in vitro findings provide valuable mechanistic insights, in vivo studies are needed to confirm whether the observed reductions in CH_4_ and shifts in fermentation profiles translate into tangible improvements in feed efficiency and environmental sustainability. Careful dose optimization is essential to balance CH_4_ mitigation with nutrient utilization, ensuring that the benefits of ML are achieved without compromising fiber digestion or protein availability. Incorporating these considerations will help guide the practical implementation of ML in ruminant diets and identify areas for future research.

## 5. Conclusions

We demonstrated that *M. oleifera* leaf extract is a promising feed additive for reducing methane production in rumen fermentation. *M. oleifera* leaf extract contains numerous active compounds with antimicrobial effects, resulting in decreased net gas production, truly degradable dry matter, and truly degradable organic matter. However, the reduction in degradability suggests a need to optimize *M. oleifera* leaf extract levels to minimize adverse effects on nutrient utilization. Further studies on various animals with different *M. oleifera* leaf extract concentrations are needed to enhance our understanding of microbial metabolic functions in the rumen under in vitro and in vivo conditions. These studies should also entail *M. oleifera* leaf extract's scalability and cost-effectiveness as a sustainable methane mitigation strategy in ruminant production systems.

## Use of AI tools declaration

The authors declare they have not used Artificial Intelligence (AI) tools in the creation of this article.
